# Bacterial Community and Anti-Cerebrovascular Disease-Related *Bacillus* Species Isolated from Traditionally Made Kochujang from Different Provinces of Korea

**DOI:** 10.3390/microorganisms9112238

**Published:** 2021-10-27

**Authors:** Gwangsu Ha, Hee-Jong Yang, Myeong-Seon Ryu, Su-Ji Jeong, Do-Youn Jeong, Sunmin Park

**Affiliations:** 1 Department of R & D, Microbial Institute for Fermentation Industry, Sunchang-Gun 56000, Korea; ksnova1492@naver.com (G.H.); godfiltss@naver.com (H.-J.Y.); rms6223@naver.com (M.-S.R.); yo217@naver.com (S.-J.J.); 2 Department of Food and Nutrition, Obesity/Diabetes Research Center, Hoseo University, Asan 31499, Korea

**Keywords:** traditionally made Kochujang, *Bacillus subtilis*, *Bacillus velezensis*, fibrinolytic activity, angiotensin I conversion enzyme inhibitory activity, antioxidant activity

## Abstract

Traditionally made Kochujang (TMK) is a long-term fermented soybean and rice mixture with red pepper and salts. The ambient bacteria in rice straw and nutrient components of Kochujang influence the bacteria community. We aimed to investigate the bacterial composition and quality of TMK from different provinces of Korea: Chungcheung (CC), Jeolla (JL), Kyungsang (KS), and GeongGee plus Kangwon (GK) provinces, and Jeju island (JJ). Furthermore, *Bacillus* spp. isolated from TMK were studied to have anti-cerebrovascular disease activity and probiotic properties. Seventy-three TMK samples from different regions were collected to assess the biogenic amine contents, bacteria composition using next-generation methods, and bacterial functions using Picrust2. *Bacillus* spp. was isolated from the collected TMK, and their antioxidant, fibrinolytic, and angiotensin I conversion enzyme (ACE) inhibitory activities and probiotic properties were examined. KS TMK had lower sodium contents than the other TMK. There were no significant differences in histamine and tyramine contents among the TMK samples in different provinces. The predominant bacteria in TMK was *Bacillus* spp., but KS included much less *Bacillus* spp. and higher *Enterococcus* and *Staphylococcus* than the other TMK. Gene expression related to lipopolysaccharide biosynthesis was higher in KS TMK than the other TMK in Picrust2. The predominant *Bacillus* spp. isolated from TMK was *B. subtilis* and *B. velezensis*. *B. subtilis* SRCM117233, SRCM117245, and SRCM117253 had antioxidant activity, whereas *B. subtilis* had higher fibrinolytic activity than other *Bacillus* spp. Only *B. velezensis* SRCM117254, SRCM117311, SRCM117314, and SRCM117318 had over 10% ACE inhibitory activity. In conclusion, KS had less *Bacillus* related to lower sodium contents than the other TMK. The specific strains of *B. subtilis* and *B. velezensis* had antioxidant, fibrinolytic, and ACE inhibitory activity, and they can be used as a starter culture to produce better quality controlled Kochujang with anti-cerebrovascular disease activities.

## 1. Introduction

In Korea, primary seasonings or sauces are based on fermented soybeans, called Jang. Jang comprises chungkookjang, doenjang, Kochujang, and kanjang. Chungkookjang is a short-term (2–3 days) fermented soybean without salts, while the other types of Jang are composed mainly of meju, fermenting cooked soybeans with or without grains for more than two months in a dry and cool condition without salts [1]. Kochujang was used as a medicinal food for promoting digestion in Hyangyak-Jipsongbang in 1433 [2]. Kochujang appeared in the diet components recorded in Siksanjip, published by Lee in the 18th century [3]. There is no similar fermented food to Kochujang using meju and red pepper powder in other Asian countries, raw chopped red pepper is added to the crushed, fermented soybeans in other Asian countries.

Unlike doenjang, mainly fermenting cooked soybeans, traditionally made Kochujang (TMK) is made of meju powder, malted glutenous rice powder, and red pepper powder, and the mixture is fermented and aged for over 6 months [4]. TMK contains higher carbohydrate sources than doenjang, and it may have diverse bacteria with high quantities. The isolated *Bacillus* spp. may have different characteristics in different types of Jang because their fermentation condition and ingredient compositions are different [5]. Functionalities of the Jang depend on the bacteria compositions and their metabolites. However, with natural fermentation it is challenging to control the bacteria community of Jang, and its quality control and functionalities are difficult to maintain. Thus, Jang fermented while inoculating specific bacteria must be examined. For example, chungkookjang fermented while inoculating specific *Bacillus* spp. has been produced and commercialized. Chungkookjang for managing glucose metabolism has been commercialized in Korea. Chungkookjang, fermented with *Bacillus* (*B.*) *amyloliquefaciens* (SRCM 100730, SRCM 100731, and SCGB 1), has probiotic properties and antidiabetic and anti-stoke activities in animal models [1,6]. Thus, TMK made with the inoculation of isolated specific *Bacillus* spp. may have a specific function. However, a few studies have isolated *Bacillus* spp. from Kochujang and assessed its functionality. These processes are crucial to make Kochujang with specific functionalities and quality control [7]. Therefore, the predominant and beneficial bacteria in Kochujang need to be isolated and the functionalities identified to make inoculated Kochujang with specific functionality.

Cerebrovascular diseases, mainly ischemic stroke, are globally increasing, associated with hyperglycemia, dyslipidemia, hypertension, and platelet aggregation [8,9]. Kochujang has been reported to control energy, glucose, lipid, and the thrombosis metabolism involved in cerebrovascular diseases in cell-based, experimental animal and human studies [4,10,11,12]. However, no studies have been conducted for the *Bacillus* spp. effect from Kochujang for anti-ischemic stroke, although some *Bacillus* spp. such as *B. subtillis* natto and other strains have antithrombotic activity [13,14]. *Bacillus* spp. from kocujang may have anti-ischemic stroke activity. Therefore, the bacteria community of traditional Kochujang needs to be analyzed, and beneficial bacteria should be isolated from Kochujang and the functionality examined for anti-ischemic stroke. Specific *Bacillus* spp. can be used to make an optimal koji inoculate for producing Kochujang with better flavor and functionalities. This study aimed to examine the bacterial community and functions of Kochujang from five different areas of Korea, and various *Bacillus* strains with inhibitory functions of cerebrovascular diseases and probiotics were isolated from the different Kochujang. The present study is novel to demonstrate that the TMK samples from different areas had different bacteria communities, and they needed better quality control. The *Bacillus* spp. having anti-cerebrovascular diseases were isolated and characterized. These results are crucial to developing inoculated Kochujang having anti-cerebrovascular diseases.

## 2. Materials and Methods

### 2.1. General Production Process of Traditionally Made Kochujang (TMK) and Sample Collection

**Figure 1:**
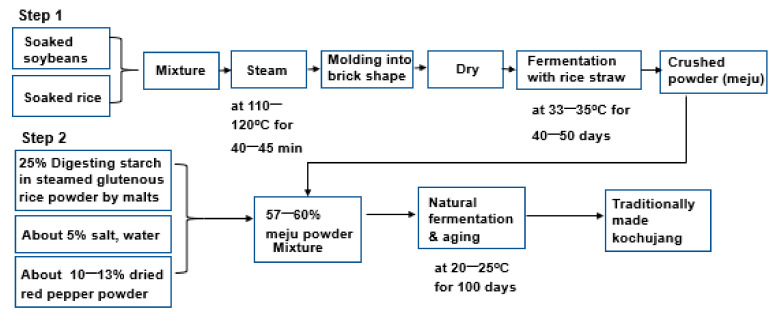
Procedures for traditionally made Kochujang (TMK).

TMK is generally made using a two-step fermentation method: (1) the fermentation of a dried brick-shape of steamed soybeans with rice straw at 33–35 °C for 40–50 days and crushed into powder (meju powder); (2) and the natural fermentation and aging of the mixture of 25% the degraded starch in glutenous rice powder with malt, 10–13% red pepper, 57–60% meju powder, and 5% salts outside at approximately 20–25 °C for over 100 days (Figure 1) [4].

**Figure 2:**
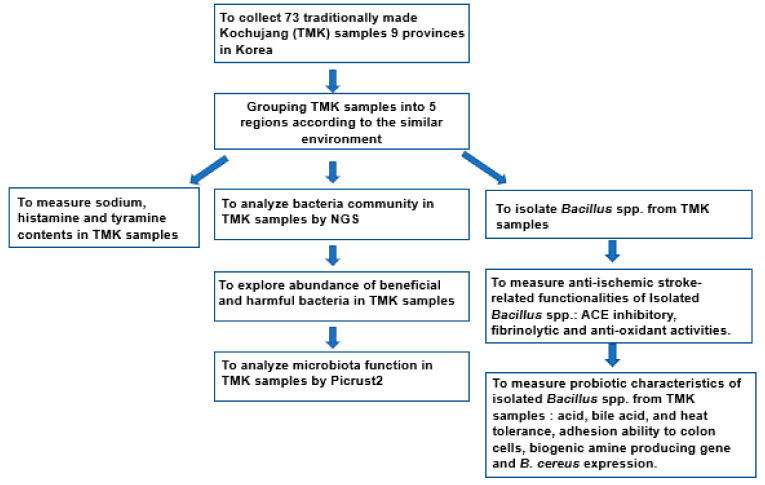
Scheme of the study design.

The scheme of the study design is shown in Figure 2. TMK samples were collected from local markets in different provinces in Korea, including Jeonbuk (14 samples), Jeonnam (nine samples), Gyungkee (seven samples), Kyungbuk (six samples), Kyungnam (six samples), Kangwon (seven samples), Chungbuk (six samples), and Chungnam (nine samples) provinces, and Jeju island (six samples). Considering the area characteristics, Jeolla (Jeonbuk + Jeonnam; *n* = 23), Kyungsang (Kyungbuk + Kyungnam; KS; *n* = 12), Gyungkang (Gyungkee-Do + Incheon + Kwangwon; GK; *n* = 17), and Chungcheung provinces (Chungbuk + Chungnam, CC; *n* = 15), and Jeju island (JJ; *n* = 6). Seventy-three TMK samples were collected in various areas of Korea in 2019–2020, and the collected TMK samples were stored at 4 °C until further analysis.

### 2.2. Sodium Contents in TMK Samples

Sodium contents of TMK samples were measured according to the Korean Food Code method 9.1.2.1.1 and 9.1.2.1.6. As sample preparation for measuring sodium contents, each TMK sample was digested using a microwave digestion system, and the digested TMK sample was diluted into deionized water. Sodium contents were measured using ICP-AES (Arcos, Spectro, Kleve, Germany) and ICP-MS (icapQ, Thermo, Waltham, MA, USA) [15].

### 2.3. Bacteria Community Analysis of the Collected TMK by the Next-Generation Sequencing (NGS) Method

The DNA extracted from the 0.2 g well-mixed TMK samples using the DNeasy PowerSoil Kit (Qiagen, Hilden, Germany) was amplified using 16S amplicon primers targeting V3 and V4 regions in a polymerase chain reaction (PCR). Libraries of the DNA PCR products were prepared according to the GS FLX plus library prep guide. The DNA was amplified with 16S universal primers in the FastStart High Fidelity PCR System (Roche, Basel, Switzerland) described elsewhere [5]. Bacterial DNA sequences in the TMK samples were measured using the Illumina MiSeq standard operating procedure on a Genome Sequencer FLX plus (454 Life Sciences, Branford, CT, USA) in the Microbial Institute for Fermentation Industry (Soon Chang, Korea).

The 16S amplicon sequences of each TMK were assessed using Mothur v.1.36. Using the Miseq SOP, the sequences were aligned using the Silva reference alignment v.12350, and the taxonomy and bacteria counts in each TMK were determined [16]. After removing the operational taxonomic units (OTUs) below 10,000 reads, the relative bacteria number of each TMK was calculated in the taxonomic assignments at the family, order, genus, and species levels. Bacteria clustering of the TMK samples from different areas was conducted by a principal component analysis (PCA) using the R package. The α-diversity with the Chao and Shannon indices was determined using the Mothur program. The counts of beneficial and harmful bacteria were analyzed in TMK samples of different areas.

### 2.4. Microbiota Function of TMK Samples

The gene functions of bacteria in the TMK samples were predicted by a PICLUST2 pipeline analysis. Among the predicted metabolic characteristics of TMK microbiota, the abundance of the Kyoto Encyclopedia of Genes and Genomes (KEGG) Orthologues was analyzed using the KEGG mapper (https://www.genome.jp/kegg/tool/map_pathway1.html (accessed on 11 March 2021)).

### 2.5. Isolation and Identification of Bacillus *spp.*

Each TMK sample (1 g) was placed in 9 mL of sterile distilled water for 30 min and serially diluted. Each diluted suspension was plated onto a Luria-Bertani (LB) agar (Difco, Sparks, MD, USA), and the inoculated agar plate was incubated at 30 °C for 24 h to separate the *Bacillus* spp. The *Bacillus* spp. in the LB plate were screened with morphological differences, and the isolates were separated for the second incubation to verify them.

Bacteria colonies were isolated from the LB plate, and their bacterial species were identified after 16S rRNA sequencing with an ABI 310 automated sequencer according to the manufacturers’ instructions (Perkin-Elmer, Foster, CA, USA), using the proper PCR primers in Macrogen Inc. (Seoul, Korea) [17]. The PCR product sequence was matched with 16S rRNA gene sequences in the NCBI GenBank database with multiple sequence alignment using the CLUSTAL W program (http://www.clustal.org/clustal2/ (accessed on 30 March 2021)).

### 2.6. Adhesion of Isolated Bacillus *spp.* into the Colon Cells In Vitro

The adhesion of *Bacillus* spp. to colon cells was conducted with the previously described method [18]. Briefly, human colon-derived CCD-18Co cells were seeded into 24-well plates at 2 × 10^5^ cells/well and incubated for 24 h. Isolated *Bacillus* spp. were inoculated to the plate at 10:1 multiplicity of infection (MOI) and incubated at 37 °C in a 5% CO_2_ atmosphere for 45 min. The cells in the wells were washed three times with fresh minimum essential media (MEM) to remove unbound bacteria by aspirating the LB media. The number of remaining bacteria was counted under a microscope, and the percentage of total bacteria added to the plate was calculated [18].

### 2.7. B. cereus and Biogenic Amine Producing Gene Expression

*B. cereus* included cytotoxin K *(CytK)*, enterotoxin FM *(ent FM)*, enterotoxin T *(bceT)*, non-hemolytic enterotoxin *(nheA)*, hemolysin BL *(hblC)*, and emetic toxin (*CER)* genes. Their gene expressions were measured in the genomic DNA of the isolated *Bacillus* spp. by PCR. When their gene expression was positive, the *Bacillus* was considered as *B. cereus* positive bacteria. Biogenic amine-producing genes, including histidine decarboxylase (*hdc*) and tyrosine decarboxylase (*tdc*), were also measured from the genomic DNA of the isolated *Bacillus* spp. by PCR. As described previously, the PCR conditions and PCR primers for *CytK, nheA, ent FM, bceT, hblC, CER, hdc,* and *tdc* were provided [19,20]. *Bacillus* spp. with decarboxylase activity was used for the positive control for *hdc* and *tdc* expression. Media with no bacteria was considered a negative control. The PCR products were identified by running electrophoresis in 2% agarose gel. The DNA bands for *CytK, nheA, ent FM, bceT, hblC, CER, hdc*, and *tdc* expression were detected [5].

### 2.8. Biogenic Amine Production and Content Measurement

Biogenic amine production by isolated *Bacillus* spp. during fermentation was determined as described previously [21]. Isolated *Bacillus* spp. was cultured in LB liquid media with 1000 ppm of tyrosine (Sigma-Aldrich, St. Louis, MO, USA) and histidine (Sigma-Aldrich) in a shaking incubator at 37 °C for 24 h. The supernatants were separated at 15,000 rpm for 30 min after incubation. Standards of histidine and tyramine were made with 0.1–100 mg/L in a 0.01 N HCl solution. The 1,7-diaminoheptane (0.1 g/L, Sigma-Aldrich) was added as an internal standard to the samples and standards (1:2, *v*:*v*) and mixed with a saturated Na_2_CO_3_ (Sigma-Aldrich) solution and 1% dansyl chloride (Sigma-Aldrich) to make derivatives.

The culture media or TMK samples were mixed with ethyl ether (Samchun, Seoul, Korea) for 3 min, and the supernatants were separated. After removing the solvent under nitrogen gas, the concentrates were dissolved into acetonitrile (Duksan, Seoul, Korea). The mixed solution was filtered with a 0.45 μm syringe filter (Sartorius, Frankfurt, Germany). The contents of biogenic amines in the culture media and TMK were then measured by a high-performance liquid chromatography (HPLC) analysis with a Cepcell Pak C18 column (2.0 × 250 mm) [21].

### 2.9. Characteristics and Cerebrovascular Disease-Related Functionalities of Isolated Bacillus

*Bacillus* spp. excrete the enzymes into the culture media when they produce the enzymes from existing genes. Extracellular enzyme activities, including angiotensin I conversion enzyme (ACE) inhibitory, antioxidant, and fibrinolytic activities, were measured with the culture media [22,23]. Each isolated *Bacillus* colony from the TMK samples was individually cultured in LB liquid media at 30 °C for 24 h, and the culture media was separated after centrifuging at 13,000 rpm for 10 min. The antioxidant activity was estimated by the 2,2-diphenyl-1-picryl-hydrazyl (DPPH, Sigma-Aldrich, St. Louis, MO, USA) activity and superoxide dismutase (SOD)-like activity [5,24]. The culture media supernatants were mixed to 100 μM DPPH in ethanol (10:1) and incubated in a dark room for 30 min. The color of the mixture was measured at 517 nm by UV/VIS spectrophotometer (SPECORD200, Analytik Jena, Jena, Germany). DPPH free radical scavenging activity (%) was calculated with the equation: (1−Absorbance of DPPH solution) × 100. The SOD-like capacity was measured with a SOD kit (Sigma-Aldrich, St. Louis, MO, USA). The culture media supernatants were incubated with a working solution of SOD kit (10:1; *v*/*v*) and mixed with a working SOD enzyme solution with the same sample volume. The mixture was incubated at 37 °C for 20 min, and the optical density was measured at 450 nm.

The fibrinolytic activity of each isolated bacteria was measured using the fibrin plate method [25]. The fibrin plate was composed of dissolving human fibrinogen in a 10 mM sodium phosphate buffer (pH 7.4) to a final concentration of 0.5%, followed by the addition of 100 unit/mL of thrombin (Sigma-Aldrich, St. Louis, MO, USA) and 1% agarose (Bio-Rad, Hercules, CA, USA). The culture media was spotted on the middle of the fibrin plate, followed by incubation at 37 °C for 24 h. When the enzymes with fibrinolytic activity exist in isolated *Bacillus*, they are secreted into the culture, and the fibrin is degraded to make a clear zone. The diameter of the cleared zone was measured [25].

The ACE inhibition activity was measured using the method reported by Ghanbari et al. [26]. The culture media of isolated *Bacillus* spp. was added to a 0.1 M sodium borate buffer (pH 8.3) and 0.5 U/mL of ACE (Sigma), and the mixture was then cultured at 37 °C for 10 min. N-Hippuryl-His-Leu hydrate (5 mM, HHL, Sigma) was added to the mixture and incubated at 37 °C for 1 h. The reaction was quenched by adding 1 M of HCl and pyridine (270970, Sigma). Benzenesulfonyl chloride (BSC, Sigma) was then added to the reaction mixture. After mixing for 1 min, it was placed on ice, and its color changes were then measured with a spectrophotometer at 325 nm. ACE inhibition was calculated using the equation, [(B − A)/(B − C)] × 100, where B is the absorbance with ACE and HHL without the ACE inhibitor component; A is the absorbance with ACE; HHL and C are the absorbance with the HHL without ACE and ACE inhibitor components, respectively.

### 2.10. Statistical Analysis

A statistical analysis was conducted using the SPSS version 20.0 (IBM Corp., Armonk, NY, USA). The results were presented as means ± standard deviations or frequency distributions. A one-way analysis of variance (ANOVA) was used to analyze the significant differences in the TMK samples according to the different regions. A Tukey’s test was conducted for multiple comparisons between groups; *p* values < 0.05 were considered significant.

## 3. Results

### 3.1. Sodium Contents and Biogenic Amine Contents

KS contained sodium contents that were significantly lower than the other TMK. The contents of biogenic amine, including histamine and tyramine, appeared to be different among the Kochujang from five different provinces. Owing to the large variations, they were similar among Kochujang samples (Table 1). The biogenic amine productions were related to the contents of *Bacillus* spp. to produce biogenic amines.

### 3.2. α- and β-Diversity of the Bacterial Community of TMK from Five Different Provinces of Korea

**Figure 3:**
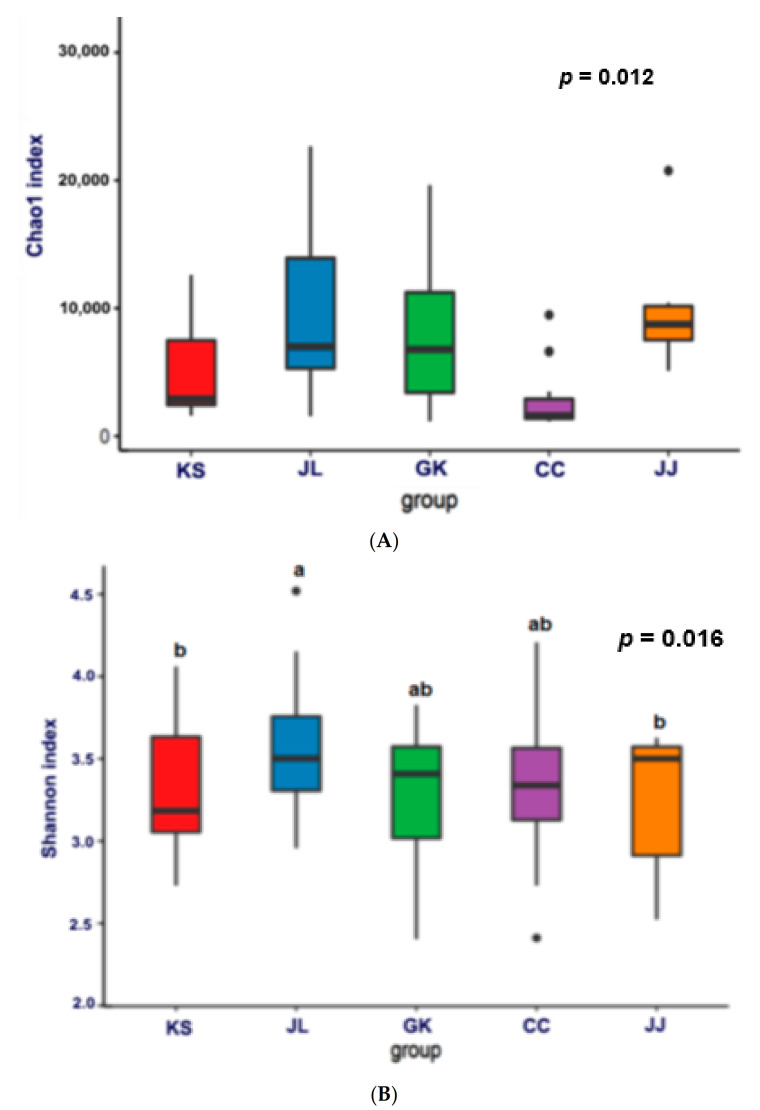

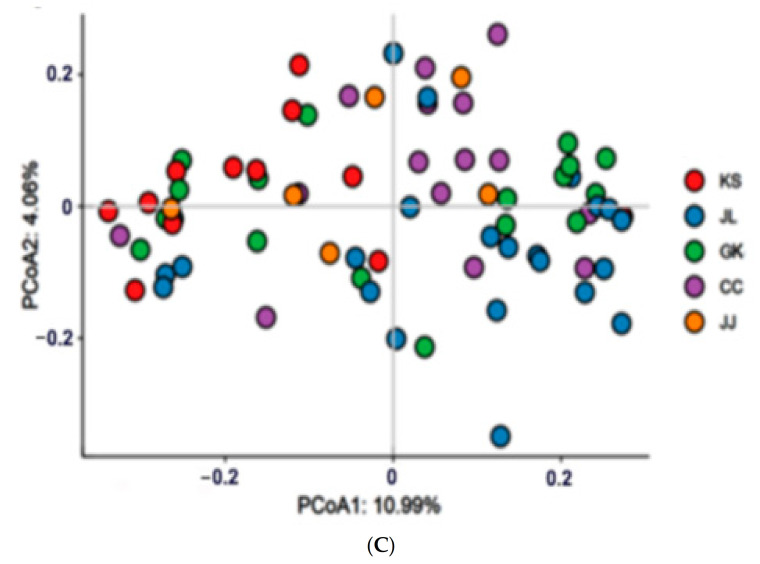
The richness of bacteria in traditionally made Kochujang (TMK). (**A**) Chao1 index for α-diversity. (**B**) Shannon index for α-diversity. (**C**) β-diversity. TMK samples from Jonlla (Chonbuk + Chonnam; *n* = 23), Kyungsang ((KS) Kyungbuk + Kyungnam; *n* = 12), Gyungkang ((GK) Gyunggi-Do + Kwangwon; *n* = 17), and Chungcheung provinces ((CC) Chungbuk + Chungnam; *n* = 15) and Jeju island ((JJ); *n* = 6) of Korea. a and b: Different letters on the bars indicate significant differences among the groups by Tukey test (*p* < 0.05).

The α-diversity was determined using the Chao1 and Shannon index representing bacterial richness in TMK. The TMK samples from JL and JJ showed the highest Chao1 index. CC had the lowest index among the groups (*p* = 0.012; Figure 3A). The Shannon index also showed a similar trend to the Chao1 index. The Shannon index of JL and JJ was higher than the other groups (*p* = 0.016; Figure 3B).

The β-diversity, a measure of similarity and dissimilarity of bacterial communities, was significantly different among the TMK of five different provinces (*p* = 0.003; Figure 3C). The bacterial communities of KS were separated from those of JL, JJ, and CC. The PCoA1 and PCoA2 axis explained 10.99% and 4.06% of bacterial diversity of all TMK samples (Figure 2C).

### 3.3. Bacterial Community of TMK from Five Different Provinces of Korea

**Figure 4:**
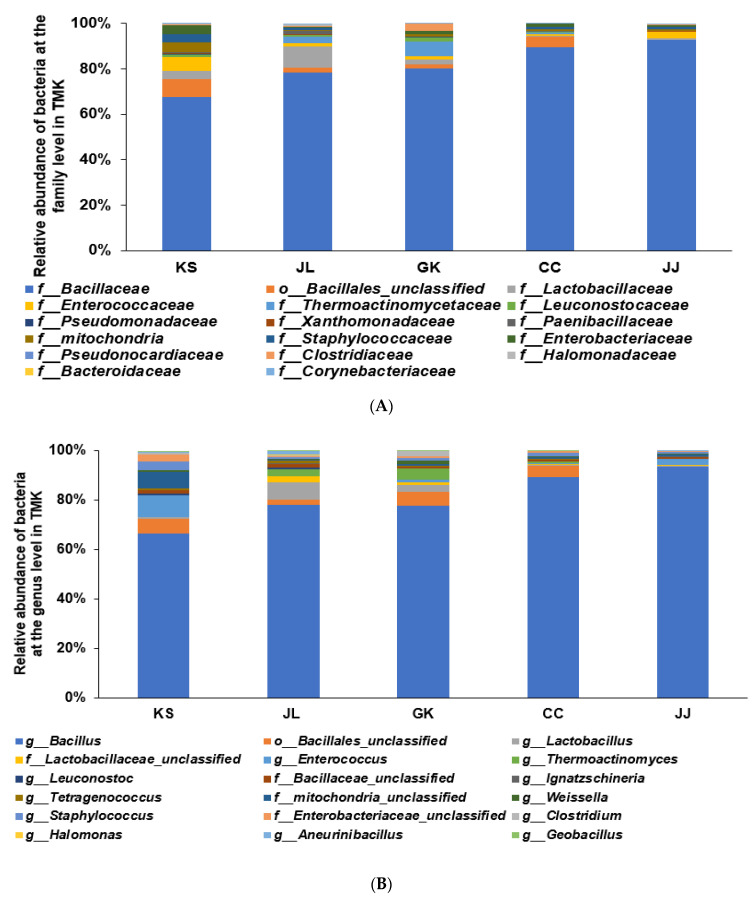

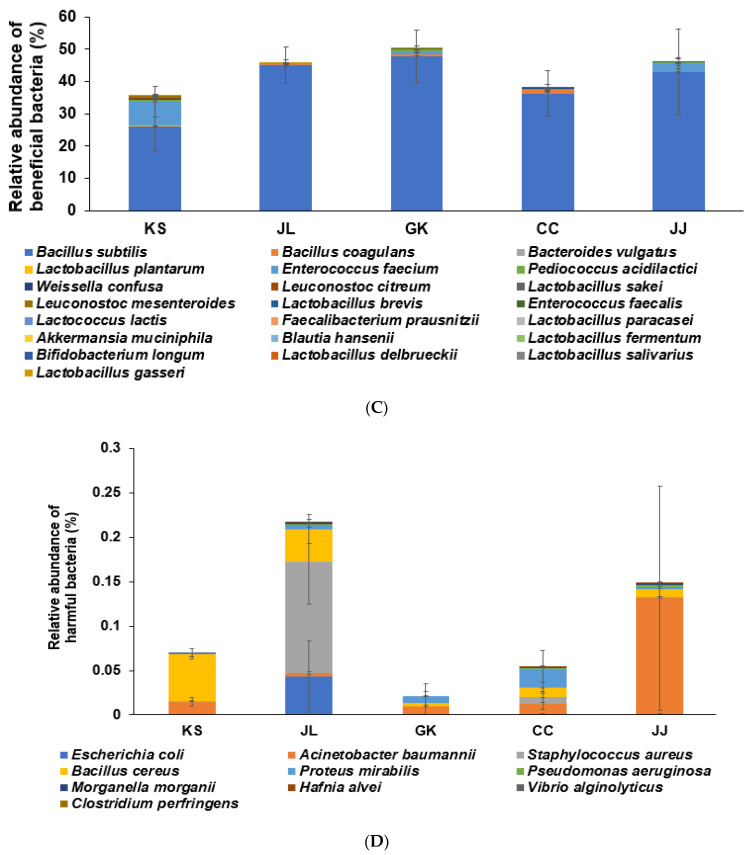

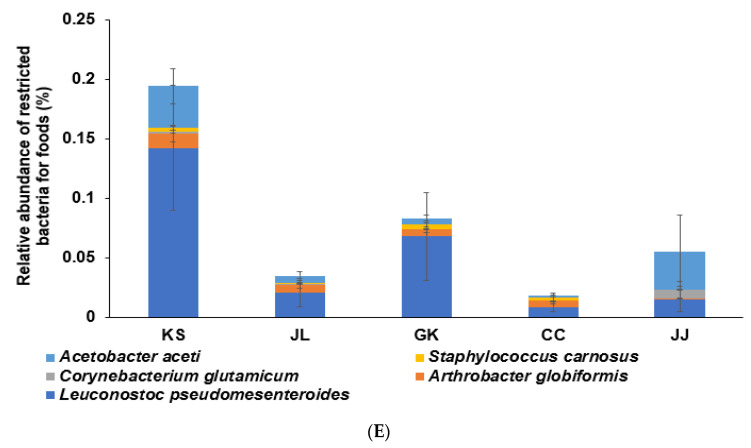
Bacterial communities of traditionally made Kochujang (TMK). (**A**) Relative abundance at the family level of bacteria. (**B**) Relative abundance at the genus level of bacteria. (**C**) Percentage of known *beneficial bacteria* at the species level. (**D**) Percentage of known *harmful bacteria* at the species level. (**E**) Percentage of restricted bacteria for food usage at the species level. TMK samples from Jonlla (Chonbuk + Chonnam; *n* = 23), Kyungsang ((KS) Kyungbuk + Kyungnam; *n* = 12), Gyungkang ((GK) Gyunggi-Do + Kwangwon; *n* = 17), Chungcheung provinces ((CC) Chungbuk + Chungnam, *n* = 15) and Jeju island ((JJ); *n* = 6) of Korea.

Among TMK from different provinces of Korea, bacterial communities of KS were different from those of other TMK samples. KS had a much lower Bacillaceae (~65%) than the other TMK in the family level, while TMK in JJ and CC had approximately 90% Bacillaceae, and JL and GK contained approximately 80% (Figure 4A). Interestingly, TMK in JL and GK contained approximately 9.3 and 4.4% Lactobacillaceae, whereas those in other provinces had less than 1% (Figure 4A). KS included much less *Bacillus* and higher *Enterococcus* and *Staphylococcus* at the genus level than the other TMK, but JL and GK contained much higher *Lactobacillus* than the TMK. CC and JJ contained more than 90% *Bacillus* (Figure 4B).

### 3.4. Beneficial Bacteria in TMK from Five Different Provinces of Korea

TMK contained 35 beneficial bacteria, and the primary beneficial bacteria were *B. subtilis* in all TMK. On the other hand, KS (26%) did not include *B. subtilis* as much as TMK from other provinces (~40%) (Figure 4C). Interestingly, KS contained 7% *Enterococcus* and *E. faecium*, but the other TMK did not include it as much. Other beneficial bacteria in TMK were *B. subtilis*, *B. coagulans*, *Bacteroides vulgatus*, *Lactobacillus plantarum, Enterococcus faecium, Pediococcus acidilactici*, and *Weissella confuse* (Figure 4C).

On the other hand, TMK contained harmful bacteria, including Escherichia coli, *Acinetobacter baumannii*, *Staphylococcus aureus*, *B. cereus*, *Proteus mirabilis*, *Pseudomonas aeruginosa*, *Morganella morganii*, and *Hafnia alvei*. JL contained the highest harmful bacteria among the TMK groups (Figure 4D). On the other hand, the mean contents of harmful bacteria in five TMK groups were less than 0.1% in all TMK groups except *Acinetobacter baumannii* in JJ and *Staphylococcus aureus* in JL TMK also contained *B. cereus* in all five TMK groups, but their contents were less than 0.1%. KS and JL contained B. cereus more than the other groups (Figure 4E). TMK included restricted bacteria for foods, including *Leuconostoc pseudomesenteroides*, *Arthrobacter globiformis*, *Corynebacterium glutamicum*, *Staphylococcus carnosus*, and *Acetobacter aceti*. KS contained restricted bacteria for foods, particularly *Leuconostoc pseudomesenteroides* and *Acetobacter aceti*, the most among the five TMK groups (Figure 4E). However, the total contents of harmful and restricted bacteria were less than 0.5% of TMK and can be acceptable for foods.

### 3.5. Metabolic Activities of the Bacteria in Area Groups of TMK by PICRUSt2 Analysis

Gene expression of bacteria in the TMK samples from the different areas showed significant differences in the relative abundance of the Kyoto Encyclopedia of Genes and Genomes Orthology (KO) involved in LPS biosynthesis, alanine, aspartate, glutamate metabolism, cofactor biosynthesis, thiamine metabolism, biotin metabolism, fructose, and mannose metabolism (*p* < 0.05; Table 2). Gene expression related to the LPS biosynthesis (*p* < 0.01) and fructose and mannose metabolism (*p* < 0.001) was higher in TMK of the KS area than that of other areas. On the other hand, gene expression related to alanine, aspartate, and glutamate metabolism was higher in TMK of JL and GK than in the other areas. TMK from KS showed lower gene expression involved in cofactor biosynthesis, thiamine metabolism, and biotin metabolism than JL and GK (Table 2).

### 3.6. Antioxidant, Fibrinolytic, and ACE Inhibitory Activities

*Bacillus* spp. with the potential activity to protect against cerebrovascular diseases were selected from the TMK. The *Bacillus* spp. can be used to make an inoculated Kochujang with anti-cerebrovascular diseases. *B. subtilis* (SRCM117233, SRCM117245, and SRCM117253) and *B. velezensis* (SRCM117254) had a high reactive oxygen species removing capacity (DPPH%) and SOD-like activity (Table 3). *B. subtilis* SRCM117233, SRCM117245, SRCM117253, and SRCM117304, *B. velezensis* SRCM117300, SRCM117301, SRCM117314, and SRCM117318, and *B. amyloliquefaciens* SRCM117311 had fibrinolytic activity with a more than 6 cm clear zone (Table 3). *B. subtilis* had high fibrinolytic activity compared with the other *Bacillus* spp. Only *B. velezensis* SRCM117254, SRCM117311, SRCM117314, and SRCM117318 had more than 10% ACE inhibitory activity (Table 3).

### 3.7. Probiotics Characteristics of Bacillus *spp.* Isolated from TMK

Table 3 lists the *Bacillus* spp. with probiotic properties. The predominant *Bacillus* spp. in TMK were *B. subtilis*, *B. velezensis*, and *B. amyloliquefaciens*. Twelve isolated *Bacillus* spp. from TMK had more than 50% survival rates at pH 2.5 and 0.3% oxgall, suggesting that 12 *Bacillus* spp. had acid-tolerance and bile salt tolerance, and more than 50% of them can go to the colon (Table 4). Interestingly, *B. subtilis* (SRCM117323) and *B. amyloliquefaciens* (SRCM117311) had 51.1 and 33.7% adhesion ability to colon cells, respectively (Table 4). Thus, they can stay in the colon to influence the host’s health.

None of the eleven *Bacillus* spp. had histidine decarboxylase (*hdc*) and tyrosine decarboxylase (*tdc*) gene expression, indicating that they did not produce biogenic amines, histamine, and tyramine (Table 4). *Bacillus* spp. was not also detected with the expression of six endotoxin genes (*CytK, nheA, ent FM, bceT, hblC, CER*) related to *B. cereus* (Table 4). The results suggest that the isolated *Bacillus* spp. did not produce *B. cereus*-related endotoxins.

## 4. Discussion

TMK is a type of Jang, which are long-term fermented soybeans in Korea. Unlike doenjang, however, it includes red pepper powder, malted grains and mainly rice. Kochujang (5%) contains less salt than doenjang (~12%) [27]. The differences in their compositions influence the bacteria communities. The present study compared the bacterial communities of 73 TMK samples from different provinces and identified the probiotic properties with anti-cerebrovascular diseases of the isolated *Bacillus* spp. KS TMK samples contained lower sodium contents than the other TMK samples, and bacterial distribution was highly different. The bacterial community must be investigated to control TMK quality by reducing harmful bacteria and biogenic amine contents and increase beneficial bacteria. Moreover, the specific *Bacillus* spp. needs to be isolated and the functionalities identified to make TMK with specific functionalities. However, a few studies have identified the bacteria community of TMK, and the functionalities of isolated *Bacillus* spp. from TMK have not been examined. The present study identified the bacterial community and metabolic function of TMK from five different areas and isolated *Bacillus* spp. having anti-cerebrovascular disease function. The results can be used to control the TMK quality in Korea, and inoculated Kochujang with an anti-cerebrovascular disease can be made and commercialized.

In Asian countries, there are several types of fermented soybean products, including natto, tempeh, douche, and sufu [28]. They are similar to chungkookjang and doenjang in Korea. In China, Doubanjiang is made by adding crushed red pepper to the broad fermented beans and wheat flour with 10–12% salts for two to three months (meju). The mixed meju and red pepper were ripened for more than 12 months. Doubanjiang is similar to Kochujang in Korea. On the other hand, its bacteria community is different from TMK. Doubanjiang contains high in proteobacteria (~50%) and Firmicutes (~40%) at the phylum level, whereas 40% of bacteria were not identified at the genus level, and the identified ones included *Bacillus* (7.5%), *Pantoea* (7%), *Halomonas* (6.4%), *Lactobacillus* (5.5%), *Sphingomonas* (5.4%), and *Staphylococcus* (5%) after a 12-month aging [29]. It indicated that the bacterial distribution was quite different between TMK and doubanjiang. The different bacterial distributions between TMK and doubanjiang might be due to the differences in the red pepper state and salt contents.

Although the dominant bacteria, *Bacillus* spp., were similar in TMK and doenjang, TMK contained more diverse bacterial communities than doenjang because of the digested rice powder and lower salt contents in TMK than doenjang [5]. TMK from different areas in Korea may have different bacterial distributions and temperatures. Unlike chungkookjang, a few studies of the TMK bacterial communities have been published, and the metabolic function of the bacteria in TMK has not been studied. The sodium contents in doenjang were reported to have a different bacterial distribution [30]. The lower salt contents (9% and 12%) in doenjang decreased the pH and increased the microbial abundance, particularly *Weissella*, *Tetragenococcus*, *Oceanobacillus*, *Debaryomyces,* and *Lactobacillus* [30]. Although the effect of salt content (5–7%) on the bacteria community has not been investigated in TMK, it may exhibit a similar impact to doenjang. The lower salt in TMK reduces *Bacillus* and promotes the diversity and abundance of bacteria when its quality is well-controlled and maintained. However, in KS TMK, beneficial bacteria were lower and harmful bacteria were higher than other TMK. JL and CK contained some *Lactobacillus*. The bacterial community differences in TMK from doenjang might be due to sodium, carbohydrate contents, and environmental temperature in the present study. Therefore, TMK can have high beneficial and diverse bacteria when its production has better control during the fermented and aging periods.

The bacterial communities of TMK in KS were separated significantly from those of TMK in other areas in the β-diversity analysis. KS contained the lowest *Bacillus* spp. (~60%). Moreover, the bacteria distribution in KS TMK was separated from the other TMK except for JJ TMK in β-diversity. Ryu et al. [7] reported that the TMK from Northern and Southern parts of KS contain *Bacillus* (54.7%), *Aerosakkonema* (23.8%), and *Enterococcus* (8.5%), which are abundant bacteria at the genus level after fermentation. Consistent with the *Bacillus* proportion in the Ryu et al. [7] study, the *Bacillus* proportion was much lower in KS TMK than the other TMK; while KS TMK, but not TMK from the other areas, contained approximately 10% *Enterococcus* and 8% *Leuconostoc*. TMK from JL and GK contained approximately 8% and 3% *Lactobacillus*. On the other hand, the bacteria composition at the species level was somewhat different between Ryu et al. and the present studies: *B. haynesii*, and *B. licheniformis* are dominant after fermentation, and *E. hirae* and *E. faecium* exist in the TMK according to Ryu et al. [7]. The present study showed that *B. subtilis* was dominant, and *B. velezensis* and *B. coagulans* were abundant in TMK.

The differences in bacterial distribution influenced the biogenic amine contents to influence the TMK quality [31]. Doenjang made with a starter culture (inoculated doenjang) has been reported to contain less biogenic amine. In the present study, biogenic amine contents were varied among TMK from different areas, and they were lower in JL than TMK from other areas. In estimating the metabolic function through Picrust2, KS TMK showed a lower LPS biosynthesis and a lower vitamin metabolism compared with TMK from other areas. However, in the present study, the biogenic amine metabolism in the Picrust2 analysis was not shown in TMK, even though gene expression of *tdc* and *hdc* was detected in some TMK. It suggests that the collected TMK did not have a high biogenic amine metabolism. In addition, a large sample size is needed to detect the bacterial distribution because the bacterial composition varies among the TMK samples. In Ryu et al. [7], regional differences in bacteria are not shown, but the result may be related to the small sample size. The sample size should be more than 10 in each province to show statistical significance. There are more than 10 samples in each area group in the present study except for JJ because Jeju is small and less varied. The present study showed that TMK from KS had a significantly different bacterial distribution from other areas.

TMK has been reported to have anti-obesity, antidiabetic, anti-dyslipidemic activities in cell-based, animal, and human studies [4,10,11,12,32]. These activities were mainly related to the metabolites of TMK containing amino acids, organic acids (citric acid), fatty acids (oleic acid and linoleic acid), sugars and sugar alcohols, flavonoids (luteolin-*C*-hexoside, quercetin-*O*-rhamnoside, and genistein *O*-dihexoside), capsaicinoids (capsaicin and dihydrocapsaicin) and capsinoids (dihydrocapsiate), and phospholipids [33]. On the other hand, few studies have examined the metabolic function of bacteria in TMK. Different bacteria in TMK make different metabolites contributing to different metabolic functions. Grains, mostly rice, are used as a carbohydrate source, but other grains are also substituted for rice. TMK with rice and wheat as koji have different antioxidant activities, and their metabolites are somewhat different [25]. The differences in metabolites are involved in growing different bacteria better in different grains. In previous studies, the predominant bacteria in Jang, including TMK, was *Bacillus*, and *Bacillus* spp. were isolated from doenjang, chungkookjang, and TMK [25]. Chungkookjang, and chungkookjang inoculated with antidiabetic activity was made from isolated *Bacillus* spp. On the other hand, some *Bacillus* spp. isolated from doenjang and TMK, have not been used to produce the inoculated doenjang and TMK.

The bacteria compositions of TMK affect its quality, including taste, odor, and metabolic functions. In the present study, some *B. subtilis* strains in TMK had higher antioxidant and high fibrinolytic activity, while some *B. velezensis* strains had more than 10% ACE inhibitory activity. Previous studies showed that tomato fermented with *B. subtilis*, sardinelle protein fermented with *B. subtilis* A26, and *B. amyloliquefaciens* An6, and *Ruditapes philippinarum* fermented with *B. natto* have shown ACE inhibitory and antioxidant activities [34,35,36]. On the other hand, few studies have examined *Bacillus* spp. to have ACE inhibitory activity. The *Bacillus* spp. to have antioxidant, ACE inhibitory, and fibrinolytic activities did not have biogenic amine and *Bacillus cereus*-related genes. They can be used to make inoculated Kochujang with anti-cerebrovascular diseases.

Overall, TMK from all areas in Korea exhibited sustainable quality control, but KS TMK must increase beneficial bacteria in the present study. TMK should maintain optimal quality control to increase various beneficial bacteria and to not contain harmful bacteria and biogenic amines. Several *B. subtilis*, *B. amyloliquefaciens*, and *B. velezensis* had anti-cerebrovascular disease activity, including increasing fibrinolytic, antioxidant, and ACE inhibitory activity. The isolated ones can be applied to make functional Kochujang as a starter culture. The inoculated Kochujang can be registered as a functional food having anti-cerebrovascular disease activity in the Korean Food and Drug Administration after its efficacy and mechanism are confirmed in animal and human studies in the near future.

## 5. Conclusions

Salt and carbohydrate contents and ambient bacteria from different areas influence the bacterial composition of TMK. TMK must be well-monitored and controlled to have good quality since it is susceptible to growing harmful bacteria due to lower salt and higher carbohydrate contents than doenjang. All TMK contained mostly beneficial bacteria and <0.5% harmful and restricted bacteria, suggesting they were well-managed. However, KS TMK may have a lower quality of bacteria than the other TMK. It is better to produce inoculated Kochujang to include well-controlled bacteria and have specific functionality. Among the isolated *Bacillus* spp. for producing inoculated Kochujang, *B. subtilis*, and *B. velezensis* had probiotic properties, no biogenic amine producing genes, antioxidant, fibrinolytic, and ACE inhibitory activities. These *B. subtilis* and *B. velezensis* strains can be used as a starter culture to make inoculated Kochujang protect against cerebrovascular diseases. Further animal and human studies are needed to produce inoculated Kochujang with optimal quality control and investigate its functionality for anti-cerebrovascular diseases.

## Figures and Tables

**Table 1:** Histamine, tyramine, and sodium contents in traditionally made Kochujang (TMK) samples from different areas of Korea.

	KS (*n* = 12)	JL (*n* = 23)	GK (*n* = 17)	CC (*n* = 15)	JJ (*n* = 6)
Sodium (%)	2.17 ± 0.19 ^b^	2.69 ± 0.09 ^a^	2.61 ± 0.14 ^a^	2.45 ± 0.14 ^a^	2.49 ± 0.30 ^a,^*
Histamine (mg/kg)	19.5 ± 12.4	22.3 ± 7.0	43.1 ± 4.7	61.1 ± 15.8	13.0 ± 5.83
Tyramine (mg/kg)	49.4 ± 26.2	27.8 ± 10.5	54.9 ± 19.9	28.1 ± 17.0	45.5 ± 31.2

Values indicated means ± standard errors. TMK samples from Jeolla ((JL), Jeonbuk+Jeonnam; *n* = 12), Kyungsang ((KS), Kyungbuk + Kyungnam; *n* = 12), Geongkang ((GK), Geonggi-Do+Incheon+Kwangwon; *n* = 17), and Chungcheung provinces ((CC), Chungbuk + Chungnam; *n* = 15) and Jeju island ((JJ); *n* = 6) of Korea. * Significantly different among the TMK from different provinces at *p* < 0.05. ^a,b^ Different letters on the bars indicate significant differences between the groups by Tukey test (*p* < 0.05).

**Table 2:** The proportion of the Kyoto Encyclopedia of Genes and Genomes Orthology values of the TMK bacterial genes involved in respective metabolism determined by Picrust2.

	KS (*n* = 12)	JL (*n* = 23)	GK (*n* = 17)	CC (*n* = 15)	JJ (*n* = 6)
LPS biosynthesis	0.15 ± 0.05 ^a^	0.03 ± 0.01 ^b^	0.04 ± 0.02 ^b^	0.03 ± 0.016 ^b^	0.01 ± 0.01 ^b,^**
Fructose and mannose metabolism	1.36 ± 0.12 ^a^	1.12 ± 0.03 ^b^	1.18 ± 0.06 ^b^	1.18 ± 0.03 ^b^	1.17 ± 0.09 ^b,^***
Alanine, aspartate, and glutamate metabolism	1.35 ± 0.02 ^b^	1.44 ± 0.01 ^a^	1.43 ± 0.01 ^a^	1.39 ± 0.01 ^b^	1.39 ± 0.02 ^b,^***
Cofactor biosynthesis	6.50 ± 0.12 ^b^	6.89 ± 0.06 ^a^	6.91 ± 0.13 ^a^	6.79 ± 0.04 ^a,b^	6.78 ± 0.09 ^a,b,^*
Thiamine metabolism	0.79 ± 0.02 ^b^	0.89 ± 0.02 ^a^	0.87 ± 0.01 ^a^	0.85 ± 0.01 ^a,b^	0.86 ± 0.03 ^a,b,^**
Biotin metabolism	0.71 ± 0.03 ^b^	0.80 ± 0.01 ^a^	0.76 ± 0.02a ^b^	0.77 ± 0.02 ^a^	0.77 ± 0.03 ^a,b,^*

Values indicated means ± standard deviations. Traditionally made Kochujang (TMK) samples from Jeolla ((JL), Jeonbuk + Jeonnam; *n* = 12), Kyungsang ((KS), Kyungbuk + Kyungnam; *n* = 12), Geongkang ((GK), Geonggi–Do + Incheon + Kwangwon; *n* = 17), and Chungcheung provinces ((CC), Chungbuk + Chungnam; *n* = 15) and Jeju island ((JJ); *n* = 6) of Korea. * Significantly different among the TMK from different provinces at *p* < 0.05, ** at *p* < 0.01, *** at *p* < 0.001. ^a,b^ Different letters on the bars indicate significant differences between the groups by Tukey test (*p* < 0.05).

**Table 3:** Functionalities of isolated *Bacillus* spp. from traditionally made Kochujang.

SRCM No.	Bacteria ID	ROS Removing Capacity (DPPH%)	SOD-Like Activity (%)	Fibrinolytic Activity (Diameter, mm)	ACE Inhibitory Activity (%)
SRCM117233	*B. subtilis*	15.9 ± 0.86	44.8 ± 2.66	68.4 ± 0.58	3.09 ± 0.55
SRCM117245	*B. subtilis*	22.3 ± 0.39	26.6 ± 3.47	80.6 ± 0.47	6.43 ± 0.29
SRCM117253	*B. subtilis*	16.5 ± 0.16	47.3 ± 1.74	77.3 ± 7.02	5.45 ± 0.67
SRCM117254	*B. velezensis*	18.1 ± 1.03	25.4 ± 4.50	38.7 ± 6.06	13.3 ± 0.00
SRCM117323	*B. subtilis*	10.7 ± 0.32	-	50.4 ± 2.77	9.96 ± 0.00
SRCM117300	*B. velezensis*	9.22 ± 0.87	-	83.6 ± 0.34	9.19 ± 0.30
SRCM117301	*B. velezensis*	14.2 ± 0.16	-	96.3 ± 2.84	6.73 ± 0.41
SRCM117304	*B. subtilis*	15.7 ± 1.90	16.5 ± 3.19	84.5 ± 11.2	3.64 ± 0.32
SRCM117311	*B. amyloliquefaciens*	3.37 ± 0.56	-	72.1 ± 0.58	20.1 ± 1.22
SRCM117314	*B. velezensis*	6.85 ± 0.71	-	92.1 ± 1.29	18.6 ± 0.19
SRCM117318	*B. velezensis*	15.9 ± 0.64	17.0 ± 4.06	76.4 ± 0.03	14.1 ± 0.37

Values represented as means ± SD (*n* = 3). No detection. ROS: reactive oxygen species; SOD: superoxide dismutase; ACE: angiotensin I converting. -, no activity.

**Table 4:** Probiotic characteristics of *Bacillus* spp. isolated from traditionally made Kochujang.

SRCM No.	Bacteria ID	Survival at pH 2.5 (%) ^1^	Survival at 0.3% Oxgall (%) ^1^	Adhesion Ability to Colon Cell (%)	Biogenic Amine-Related Genes ^2^	*B. cereus* Related Gene Expression ^3^
SRCM117233	*B. subtilis*	67.1 ± 2.43	76.3 ± 0.75	-	-	-
SRCM117245	*B. subtilis*	63.7 ± 1.10	64.3 ± 0.91	-	-	-
SRCM117253	*B. subtilis*	62.8 ± 1.46	65.2 ± 1.43	-	-	-
SRCM117254	*B. velezensis*	83.6 ± 2.20	84.2 ± 1.39	-	-	-
SRCM117323	*B. subtilis*	105.5 ± 5.27	96.2 ± 3.94	51.1 ± 0.92	-	-
SRCM117300	*B. velezensis*	54.3 ± 0.80	51.5 ± 1.58	-	-	-
SRCM117301	*B. velezensis*	62.8 ± 2.73	59.7 ± 1.10	-	-	-
SRCM117304	*B. subtilis*	112.0 ± 1.77	68.5 ± 0.48	-	-	-
SRCM117311	*B. amyloliquefaciens*	89.5 ± 1.56	89.8 ± 1.17	33.7 ± 2.45	-	-
SRCM117314	*B. velezensis*	68.9 ± 1.29	62.8 ± 2.12	-	-	-
SRCM117318	*B. velezensis*	90.6 ± 0.73	75.8 ± 1.22	-	-	-

Values represented as means ± SD (*n* = 3). ^1^ The percentage of the number of live cells after the reaction and before the reaction. ^2^ Determined by histidine decarboxylase (hdc) and tyrosine decarboxylase (tdc) expression. ^3^ *CytK, nheA, ent FM, bceT, hblC, CER* gene expression related to *B. cereus*. -, no activity.

## Data Availability

Data are available from the corresponding author on reasonable request with a reasonable reason.
